# Naloxone Alleviate the Severity of Delirium in Hospitalized Patients With Parkinsonism: Three Case Reports

**DOI:** 10.3389/fpsyt.2021.748958

**Published:** 2021-10-27

**Authors:** Haiyan Jin, Jie Zhang, Qiongyue Hu, Junjiao Ping, Tingyun Jiang, Baoguo Du, Xin Duan

**Affiliations:** ^1^Department of Psychiatry, Ruijin Hospital, Shanghai Jiaotong University School of Medicine, Shanghai, China; ^2^Department of Psychiatry, The Third People's Hospital of Zhongshan, Zhongshan, China; ^3^Department of Psychiatry, Qingdao Mental Health Center, Qingdao University, Qingdao, China; ^4^Department of Geriatric Psychiatry, Wuzhongpei Memorial Hospital, Foshan, China

**Keywords:** naloxone, delirium, Parkinson's disease, parkinsonism, anticholinergic drugs, anti-PD medications

## Abstract

**Purpose:** Delirium is common in geriatric with Parkinson's disease (PD). Treatments for delirium have generally been neuroleptics; however, antipsychotics have potential effect to block striatal dopamine D2 receptors and worsen symptom of parkinsonism. We explored whether naloxone can alleviate delirium in PD and other forms of parkinsonism.

**Patients and Methods:** Patients with parkinsonism who met the delirium criteria of the Diagnostic and Statistical Manual of Mental Disorders, Fifth Edition (DSM-5) received naloxone infusions once or twice daily. Treatment effects were evaluated by the delirium rating scale–revised 98 (DRS-R98), including non-cognitive and cognitive subscales; the Richmond agitation–sedation scale (RASS); and the mini mental status examination (MMSE).

**Results:** Two patients with primary parkinsonism, one with vascular PD were observed. The daily dose of naloxone was 2.08 ± 0.64 mg (range: 1–4 mg). Medication time last from 1 h to 7 days without side effects observed. Following with naloxone infusions, DRS-R98 scores decreased within 12 h and MMSE scores increased. The psychotic symptoms, disorientation, and attention deficits were alleviated significantly, while RASS scores decreased with naloxone treatment.

**Conclusion:** Naloxone alleviated psychotic symptoms, improved cognitive dysfunction, and irritability in patients with delirium in the context of PD. The preliminary findings point out that the opioid system may be involved in the pathophysiology of delirium, which may be one of potential treat targets for delirium of PD.

## Introduction

Nigrostriatal dopamine deficiency is well-recognized as the primary biochemical abnormality in Parkinson's disease (PD). Delirium is common in geriatric with PD, particularly in those receiving anticholinergics and dopamine agonists (1), and increases the risk of dementia, motor impairment, and mortality (2). The pathophysiology of delirium in PD is poorly understood. Neurotransmitter imbalances, such as loss of acetylcholine and/or an excess of dopamine, are thought to be common pathways (3–5). To date, first-line treatments are neuroleptics for delirium, such as haloperidol. Quetiapine is recommended with the fewest side effects in PD (6). However, antipsychotics except pimavanserin have the potential effect to block striatal dopamine D2 receptors and thus worsen motor features in parkinsonian, and all of them are considered relating to increase mortality in elderly dementia patients (7). The U.S. Food and Drug Administration (FDA) has not approved any agents for delirium treatment.

Researchers found that naloxone enhanced the release of acetylcholine (8, 9) and reduced the content of dopamine (10) in specific cerebral areas in rats. Other studies reported that naloxone could improve spatial working memory impairments by induced scopolamine in animals (11). It also was reported to alleviate or eliminate auditory and visual hallucinations in individuals with schizophrenia (12, 13). Based on known findings, we speculated that naloxone could improve delirium in PD. Delirium is characterized with cognitive defects, including memory impairments, and psychotic symptoms such as hallucinations. We present a case series of three patients with parkinsonism followed up 2 years, they were treated by naloxone infusion for delirium.

## Materials and Methods

The patient's demographics, concomitant diagnosis, clinical profile, and naloxone use are summarized in Table 1. Efficacy of naloxone was evaluated daily during delirium episodes using the delirium rating scale–revised 98 severity scale (DRS-R98) and the Richmond agitation–sedation scale (RASS). Cognitive status was assessed by the mini mental status examination (MMSE) before and after naloxone treatment. All assessments were blinded. Informed consent was obtained from the guardians of all patients, and the ethical issues were approved by the Ethics Committee of the hospital.

**Table 1:** Patients' demographics, clinical profile, and naloxone usage.

**Patient**	**Age/Sex**	**Medical diagnosis**	**Natural course of delirium** **(days)**	**Symptoms and signs before naloxone**	**Lab examination and brain CT or PET**	**Naloxone protocol**	**Delirium duration after naloxone (days)**
1	66/M	Parkinson's disease, right side PVP, dementia	20	Distractibility, simple, and irrelevant answers to questions, slurred speech, disorientation, persecutory delusion, bradykinesia, postural instability, tremor, and increased muscle tone in RUE.	Serum electrolytes, renal and liver functions within normal limits; CT, cerebral atrophy; following changes in skull and lobe after right side PVP.	NS 250 ml + naloxone 2 mg IV drop in morning on day 1; the drug was withheld in evening owing to somnolence; administered twice on day 2; withheld on day 3 owing to oversedation; reinstated nightly on days 4–8.	<6/24 (the first intervention) <12/24 (the second intervention)
2	76/F	Vascular parkinsonism, vascular cognitive impairment, hypertension	7	Lags in response, disorientation in time and place, anxiety, hallucinations, bradykinesia, unstable gait, right knee tendon reflex (+++), bilateral Babinski sign (–).	Serum electrolytes, renal and liver functions within normal limits; CT (16 days before admission): left basal ganglia and thalamus lacunar infarction.	NS 250 ml + naloxone 2 mg IV drop at night on days 1–5.	12/24
3	56/F	Parkinson's disease, depression without psychotic symptoms	2/24	Lags in response, pulling at bedsheets or clothes, time perception errors, disorganized speech, dilated pupils, decreasing limb and body rigidity, improving dyskinesia.	Serum electrolytes, renal and liver functions within normal limits; PD confirmed by PET at 2 week follow-up.	NS 100 ml + naloxone 1 mg IV drop at noon.	1/24

*M, male; F, female; PVP, posteroventral pallidotomy; RUE, right upper extremity; NS, normal saline; CT, computerized tomographic scan; PET, positron emission tomography*.

## Results

Delirium duration (7.75 ± 5.32, range: 1–12 h) (the patient 1 including two treatment intervals) was much shorter than the natural course of delirium (9.03 ± 10.11, range: 0.08–20 days) after naloxone intervention in three patients. Thirteen domain scores of the DRS-R98 severity scale indicated that perceptual disturbances, the hallucinations (2.75 ± 0.50) and sleep-wake cycle disturbances (2.22 ± 1.41) were more prominent problems in view of non-cognitive subscale; long-term memory impairment and visuospatial impairment (both 2.25 ± 0.95) were more severity in view of cognitive subscale before naloxone intervention. Compared to pre-treatment, the occurrence of perceptual disturbances and hallucinations (2.75) significantly descended followed by sleep-wake cycle disturbances (2.22), affective labilities, long-term memory impairment and visuospatial impairment (2.00, respectively), delusions (1.88), thought process abnormalities, motor agitation, orientation problems, and attention deficits (1.75, respectively). The scores are shown in Table 2. The daily dosage of naloxone was 2.08 ± 0.64 mg (range: 1–4 mg). The mean duration of naloxone treats was 4.01 ± 3.58 days (range: 1 h−7 days). We observed that naloxone was safe and well-tolerated in all three patients.

**Table 2:** Baseline DRS-R98 scores and changes after naloxone treatment (*N* = 3).

**Item of DRS-R98**	**Pre-treatment** **Mean ± SD**	**Post-treatment** **Mean ± SD**	**Change difference**
(1) Sleep-wake cycle disturbances	2.22 ± 1.41	0	2.22
(2) Perceptual disturbances and hallucinations	2.75 ± 0.50	0	2.75
(3) Delusions	1.88 ± 1.44	0	1.88
(4) Affective labilities	2.00 ± 0.82	0	2.00
(5) Language problems	1.38 ± 0.95	0	1.38
(6) Thought process abnormalities	1.75 ± 0.50	0	1.75
(7) Motor agitation	1.75 ± 0.29	0	1.75
(8) Motor retardation	0	0.75 ± 1.50	0.75
(9) Orientation problems	1.75 ± 0.50	0	1.75
(10) Attention deficits	1.75 ± 0.50	0	1.75
(11) Short-term memory impairment	1.50 ± 1.00	0.25 ± 0.50	1.25
(12) Long-term memory impairment	2.25 ± 0.95	0.25 ± 0.50	2.00
(13) Visuospatial impairment	2.25 ± 0.50	0.25 ± 0.50	2.00

*DRS-R98, delirium rating scale–revised 98 severity scale; SD, standard deviation*.

### Patient #1

This patient was a 66-year-old man, who was diagnosed with PD 8 years previously. For the past 2 years, he had been taking 100 mg/400 mg of benserazide/levodopa and 2 mg of benzhexol four times a day. However, the disease continued to worsen, so entacapone (100 mg) was added twice daily. Twenty days ago, he began to experience hallucinations that thieves were hiding on the second floor of his house, he once hurriedly jumped into a pool in front of his house to chase them. His confusion and behavioral dysfunction were most serious at night, with severe insomnia. According to DSM-5, he was diagnosed with persistent hyperactive delirium; the delirium was due to PD and anti-PD medications.

**Figure 1:**
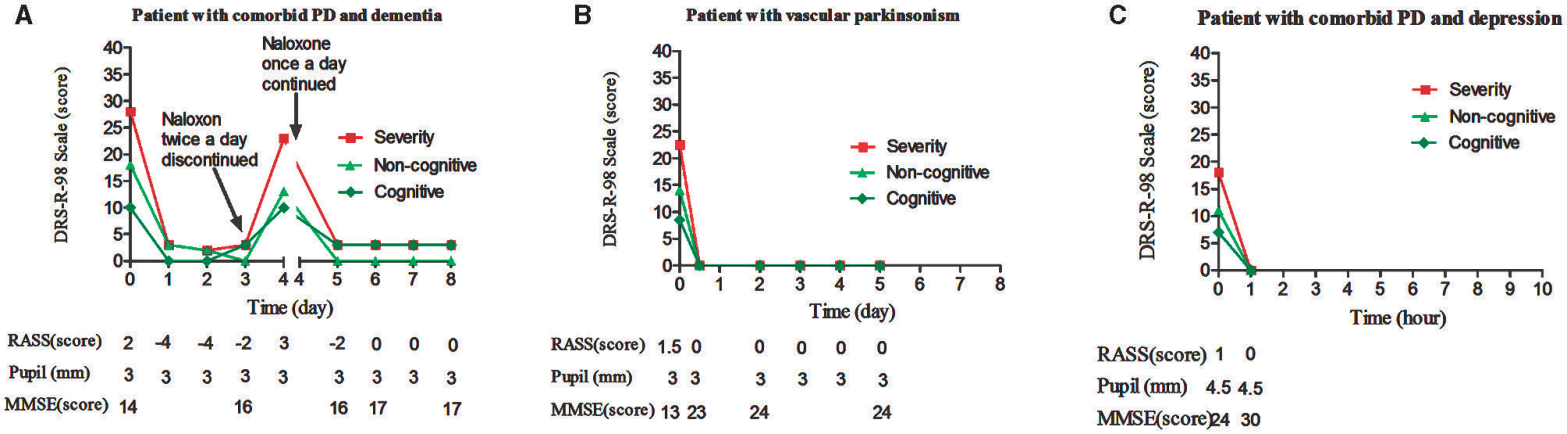
Change in DRS-R98 scores over the study period, and corresponding measurements. **(A)** DRS-R98 severity and RASS scores decreased during each of the two treatment intervals. In the first, under twice-daily naloxone, the RASS was −4, indicating the agitated patient was over sedated on days 1 and 2. The patient was delirium-free and mildly sedated on day 3 without naloxone treatment. On day 4, DRS-R98 severity and RASS scores were 23 and 3, respectively, indicating that delirium had returned. In the second interval, under nightly naloxone only, the patient was delirium-free and mildly sedated on day 5. MMSE scores were 14–16 and stabilized at 17 with remaining cognitive deficits. Corresponding pupil diameter was normal. **(B)** DRS-R98 severity and RASS scores decreased over time. A change in RASS score from 1.5 to 0 indicated that the mildly agitated patient became calm after sleeping 12 h at night. The MMSE score increased from 13 to 23 and stabilized at 24 with remaining cognitive deficits. Corresponding pupil diameter was normal. **(C)** DRS-R98 severity and RASS scores decreased over time. RASS score of 1–0 indicated that the patient recovered from a restless to a normal state. The MMSE score increased from 24 to 30 indicating no cognitive deficits; however, pupil diameter remained 4.5 mm (normally 3 mm). DRS-R98, delirium rating scale-revised 98 (severity scale only; 13 items; maximum score, 39; delirium, >15; resolution, <12); MMSE, mini mental status examination (30 point cognitive test; maximum score 30); RASS, Richmond agitation sedation scale (10 levels; 4, combative; 3, very agitated; 2, agitated; 1, restless; 0, alert and calm; −1, drowsy; −2, light sedation; −3, sedation; −4, deep sedation; −5, cannot be aroused).

Benzhexol was discontinued, and benserazide/levodopa was reduced to three times with concomitant of entacapone twice a day. On the night of admission, he was irritable and refused to take medications. The next day morning, the patient reported two thieves were running after him during the night, then naloxone was medicated (Figure 1A). The treatment caused the patient somnolence whole day, while psychotic symptoms were not detected on day 1 and next day. aloxone was withheld on day 3 and the patient regained full orientation and showed apparent improvement in delayed recall. However, on the afternoon of the fourth day, he suddenly tore up a box and threw it, and poured water on the bed. He was given a second round of naloxone treatment, and became calm and fell asleep quickly that night. On the morning of the fifth day, he regained clarity of awareness, but still displayed static tremor and bradykinesia. He explained that the previous night he had feared that he was surrounded by about 20 villains who wanted to rob him. Thereafter, naloxone treatment was maintained for 3 days without the recurrence of delirium. There were not antipsychotics or benzodiazepines used in all processes.

During 3 months of follow up, the patient remained functionally and mental stable with combination of 50 mg/200 mg of benserazide/levodopa three times a day and 200 mg of entacapone twice a day.

### Patient #2

The patient was a 76-year-old woman diagnosed with secondary vascular PD after a cerebral infarction 6 years previously. She had been taking 50 mg/200 mg of benserazide/levodopa and 100 mg of amantadine three times a day, in combination with 20 mg piribedil twice a day. Her essential hypertension was controlled by nifedipine with normotension. One week before admission, the woman began to manifest disturbances of awareness, composed of vivid, horrible, or absurd auditory and visual hallucinations that were richer at night. Her symptoms were more serious in the 3 days before admission, with insomnia almost whole night. Her diagnosis was persistent hyperactive anti-PD medication-induced delirium. Amantadine and piribedil were stopped, while benserazide/levodopa was continued. In addition, naloxone was administrated at night (Figure 1B). Her delirium vanished completely just after a whole night's good sleep, which notably improved her psychotic symptoms, orientation, and delayed recall. Naloxone was maintained for 5 days and there was no recurrence of delirium. No concurrent interventions were applied.

The patient remained mental stable after 3 months with the same dose of benserazide/levodopa.

### Patient #3

A 56-year-old woman began suffering from fatigue and muscle soreness 6 years previously, leading to irritation, anxiety, and occasional suicidal ideation. The diagnosis was presumed to be PD and depression, but her symptoms sometimes worsened with adjusting dose of benserazide/levodopa and occasional fluoxetine. She was restless, and then admitted to our hospital. For the first 3 weeks of hospitalization, the patient received 20 mg of paroxetine with benserazide/levodopa (150 mg/600 mg), carbidopa/levodopa (100 mg/400 mg), and 100 mg of entacapone daily. However, the patient's symptoms remained refractory. Her physician regarded the depressive symptoms as the side effect of levodopa, so anti-PD medications were tapered to stopped. Paroxetine was continued and increased to 40 mg/day. Consequently, the patient developed a lumbering gait with stiffness in the limbs. Then this status was thought to be paroxetine-induced parkinsonism, so the paroxetine dose was halved and supplemented with 150 mg of venlafaxine in prolonged-release capsules, 1.5 mg of lorazepam, and 0.8 mg of alprazolam daily. The patient's parkinsonism worsened. Benzhexol (2 mg) was then used twice daily for 2 days in combination with 0.3 mg scopolamine in order to reduce muscle tone.

Unfortunately, although the patient's muscle tension relieved, she developed a clouding of consciousness characterized by impaired time perception, disorganized speech, and uncontrolled limb movements. She sometimes babbled “porridge on the mosquito net,” or ordered her family to open the door or buy vegetables. She was diagnosed with acute hyperactive delirium; delirium due to PD and anticholinergic drugs. Naloxone infusion was carried out (Figure 1C). Her delirium was resolved after 1 h of naloxone treatment, which also visibly improved deficits of immediate and delayed recall, although her pupil diameter remained 4.5 mm. During the course of naloxone infusion, she didn't receive any other medications. Over the next 2 days, the patient was able to walk around the ward. After that, her Parkinson's symptoms reappeared with muscle rigidity, bradykinesia, and hyperreflexia, and a diagnosis of PD with depression was confirmed.

Follow-up treatment used a combination of benserazide/levodopa (150 mg/600 mg) and paroxetine (40 mg) with no anticholinergic drugs, and her delirium did not recur.

## Discussion

We observed that naloxone improved dramatically delirium in three hospitalized patients with primary PD and secondary vascular PD. The severity of delirium was alleviated rapidly, determined with simultaneous decreases in DRS-R98 scores in both the non-cognitive and cognitive domains. Psychotic symptoms, disorientation, and attention deficits were the first and most symptoms to be improved obviously, and patients became noticeably calmer.

In patient 1 and 2, concomitant use of dopamine preparation and/or dopamine agonists and anticholinergic drugs contributed to the development of delirium (1). Both PD and cognitive decline may be important risk factors for delirium (6, 14). We emphasized that the management of anti-PD-medication-induced delirium is keeping a balance between reducing the causative agent and maintaining motor function. However, we believe that naloxone was associated with the rapid delirium resolution as we observed. First, there was a rapid onset of sedation and the elimination of psychosis in two patients. Second, the dosage of 2 mg twice a day caused over-sedation in patient 1 on initial 2 days, so naloxone dosage had to be halved on the second intervention. These results indicate that naloxone has a dose-dependent effect on delirium, because naloxone itself has not pharmacological sedative effect. Third, premature discontinuation of naloxone caused delirium relapsing in patient 1. It suggested it is necessary to maintain a full course of treatment with naloxone. In this study, various dose of benserazide/levodopa was managed individually for patients involved, since the parkinson's symptom was required to be managed at stable level.

Inouye and colleagues observed that poly-pharmacy is a risk factor for delirium (15). The combination of scopolamine and benzhexol with paroxetine, venlafaxine, and benzodiazepines may have played a role for third patient's delirium. Paroxetine has potent anticholinergic effect, which can increase the risk of delirium. Anticholinergic drugs are believed to play a fundamental role in delirium (16, 17). However, we consider that resolution methods for delirium unlikely were consequence of attenuation to the effect of anticholinergics. We think naloxone playing a crucial role in delirium resolution, because the patient's symptoms of delirium disappeared after 1 h treatment, but the effects of anticholinergic agents remained, it is verified from her pupils remaining enlarged and locomotion continually improved.

The acetylcholinergic neurotransmitter system is key for the function of alertness, attention, memory, and learning in the central nervous system. Hypocholinergia results in the hallmark “clouding of consciousness” and inattention seen in delirium. Elevation of dopamine can result in frank hallucinations, which is one of causes of agitation in the delirious patient (18, 19). Similar to shown in animals, in patients with delirium, we presumed that naloxone could synergistically complement neurotransmitter imbalances, promoting the release of acetylcholine (8, 9) to improve orientation, attention, and memory impairment, and limiting the increase in dopamine (10) to reduce psychotic symptoms.

Interestingly, naloxone had a rapid-onset effect on sedation or sleep ameliorated in three patients. These findings is consistent with Berger and colleagues' case report, in which Mr. A became “clearly less agitated” after naloxone injection (12), and is also consistent with our previous finding that naloxone could control agitated behavior in delirium after a stroke (20). Accumulating evidences indicate that all three opioid receptors (mu, delta, and kappa) mediate the mechanisms associated with emotional responses and mood control (21). Particularly, naloxone has been proved to active the mu receptor, which in turn interact with dopaminergic and acetylcholine system facilitating neurotransmitter releasing to alleviate cognitive and psychotic symptom (9, 10, 22). This phenomenon suggests a causal relationship with the gamma-aminobutyric acid (GABA)ergic system (23, 24). Agitation in delirium is very complex that needs to be addressed. Some previous studies have reported that antipsychotics such as haloperidol contribute mainly to sedation in the treatment of delirium (25, 26).

Notably, naloxone intervention was free of side effects in our patients, so we could avoid anticholinergic effects and extrapyramidal symptoms that occur frequently on antipsychotics treatment.

There are several limitations in this observational study. The three cases involved in the pilot study limited the statistical power for validity of conclusion. Poly-pharmacy in some cases involved and uncertain neurobiological mechanism make it difficult to explain the efficacy of naloxone on delirium occurred with patients with PD. Few rating scales involved limited the efficacy for evaluating the multiply domains in cognitive functioning for patients in the study. This case series highlighted the clinical significance that naloxone contributed potentially to manage the symptoms in delirium of patients with PD.

The research gives insight into why naloxone may be a promising agent for the treats of delirium in the context of PD. However, we think further pharmacological studies on how effects of naloxone on brain neurotransmitter in patients with delirium are necessary to prove this observation.

## Conclusion

Naloxone was safe and well-tolerated in our studies. It alleviated psychotic symptoms, improved cognitive dysfunction, and calmed irritability in patients with PD delirium. The results imply that the opioid system is perhaps related to complex interactions between dopamine, acetylcholine, and GABA, which implicate in underlying pathophysiology of delirium. Further observations need provide pilot data to support a double-blind, placebo-controlled clinical trial to demonstrate the efficacy of naloxone in the treatment of delirium in PD and parkinsonism.

## Data Availability Statement

The raw data supporting the conclusions of this article will be made available by the authors, without undue reservation.

## Ethics Statement

The studies involving human participants were reviewed and approved by Ethics Committee of the Shunde Wuzhongpei hospital. The patients/participants provided their written informed consent to participate in this study. Written informed consent was obtained from the individual(s) for the publication of any potentially identifiable images or data included in this article.

## Author Contributions

HJ and JZ: concept and design, data acquisition, analysis and interpretation, statistical analysis, and drafting the manuscript. QH: data acquisition, analysis, and statistical analysis. JP: critical revision of the manuscript. TJ: data analysis. BD: data interpretation and supervision. XD: concept and design and critical revision of the manuscript. All authors read and approved the final manuscript.

## Funding

This study was supported by Foshan Scientific and Technological projects (200808112), the grants from Shanghai Municipal Health Commission (2019ZB0201), and Social Welfare Scientific Research Project (Key Program) of Zhongshan City (2018B1006). This research received no specific grant from any funding agency in the public, commercial, ornot-for-profit sectors.

## Conflict of Interest

The authors declare that the research was conducted in the absence of any commercial or financial relationships that could be construed as a potential conflict of interest. The Handling Editor ZL declared a shared affiliation, though no other collaboration, with one author HJ at the time of the review.

## Publisher's Note

All claims expressed in this article are solely those of the authors and do not necessarily represent those of their affiliated organizations, or those of the publisher, the editors and the reviewers. Any product that may be evaluated in this article, or claim that may be made by its manufacturer, is not guaranteed or endorsed by the publisher.
